# Meeting report of the 4th autumn meeting of the German Society of Extracellular Vesicles (GSEV): cutting edge EV research driven by young scientists

**DOI:** 10.20517/evcna.2021.23

**Published:** 2021-12-30

**Authors:** Eva-Maria Krämer-Albers, Elke Pogge von Strandmann, Gregor Fuhrmann, Irina Nazarenko, Bernd Giebel, Michael W. Pfaffl

**Affiliations:** ^1^Institute for Developmental Biology and Neurobiology (IDN), Cellular Neurobiology, Extracellular Vesicles, Mainz 55128, Germany.; ^2^Philipps-Universität Marburg (PUM), Institute for Tumor Immunology, Center for Tumor Biology and Immunology (ZTI), Marburg 35043, Germany.; ^3^Friedrich-Alexander-University Erlangen-Nürnberg (FAU), Pharmaceutical Biology, Department of Biology, Erlangen 91058, Germany.; ^4^Institute for Infection Prevention and Hospital Epidemiology; Medical Center - University of Freiburg, Faculty of Medicine, University of Freiburg, Freiburg 79106, Germany.; ^5^Institute for Transfusion Medicine, University Hospital Essen, University of Duisburg-Essen, Essen 45122, Germany.; ^6^Animal Physiology and Immunology, Technical University of Munich, School of Life Sciences, Freising 85354, Germany.

## INTRODUCTION

The German Society for Extracellular Vesicles was delighted and excited to conduct its yearly autumn meeting on October 2nd and 3rd 2021 in Freiburg/Breisgau, a charming university town at the foot of the Black Forest. Organized by Irina Nazarenko and her team, it was one of the first in person meetings in Germany since the start of the pandemic in accordance with current coronavirus safety regulations in the beautiful Paulus Saal in the center of Freiburg old town. Although GSEV held one of the last possible in person meetings in March 2020 (IGLD/GSEV Meeting in Frankfurt) as well as conducted a virtual meeting in autumn 2020 (organized by Gregor Fuhrmann, Saarbrücken), it felt like a long time not having interacted. Consequently, the 150 participants felt a vibrant atmosphere and the air of excitement, having the opportunity to meet again in person and discussing the latest developments in extracellular vesicle (EV) science.

The GSEV autumn meeting traditionally has the intention to actively involve and bring into focus EV scientists at the early career stage and to drive interactions at the current frontiers of EV research. To this end, five keynote lectures of invited internationally renowned EV scientists were combined with 25 presentations of young scientists selected from the abstracts and 9 presentations of technological innovations provided by industrial sponsors. The program was framed by two poster sessions (displaying 27 posters), an industrial exhibition, a social event with Mexican folklore in the evening, and a final round table discussion on the value of the Minimal Information for Studies of Extracellular Vesicles (MISEV) guidelines and pitfalls of EV research. The best two presentations and three posters were awarded with the ***Margot Zöller Prize*** and ***Peter Altevogt Prize***, respectively. These two renowned researchers, who pioneered the EV research in Germany, enthusiastically sent short encouraging messages to the prize winners and the audience. Thanks to all participants, the organizing team, and the supporting stuff, the meeting provided an enjoyable, lively atmosphere, the perfect prerequisite for productive discussions. In the following, we report on the six scientific sessions and summarize their main scientific content including topics of discussion.

## EV ORIGIN AND BIOGENESIS

The scientific program of the meeting started with a session on EV origin and biogenesis chaired by Eva-Maria Krämer-Albers (Mainz, Germany) and Melanie Schwämmle (Freiburg, Germany). The kick-off keynote lecture was given by Graca Raposo (Institut Curie, Paris, France), who took the audience on a journey through the history of EV science, from its roots to the most recent findings of her group on EV functions in development. Raposo^[1]^ pioneered the field in the 1990s by her discovery that B cell-derived EVs carry MHC-class II and present antigens that can activate T cells. This discovery showed for the first time that EVs can deliver signals to other cells and, thus, paved the way for the whole field. More recently, her team found that EVs in the skin mediate bidirectional communication between melanocytes and keratinocytes regulating skin cell differentiation, pigmentation, and homeostasis^[2]^. Moreover, the team used *Drosophila* as a model to explore EVs shedding from epithelial microvilli *in vivo*. Engaging the protein Prominin, these small EVs apparently bud from microvilli and are loaded with Hedgehog, essentially being involved in the development of the *Drosophila* wing imaginal discs. Finally, Graca took the opportunity to dedicate her presentation to the 80th birthday of Philip Stahl, who together with Harding and in parallel to Johnstone discovered the exosome secretion pathway in maturing reticulocytes^[3,4]^. Although EV structures had been mentioned before in the literature, these discoveries initiated and mark the beginning of modern EV research.

The session continued with selected presentations from early career researchers. Wolf (Paracelsus Medical University, Salzburg, Austria) presented new work on the protein corona, which appears to form on the surface of EVs according to their biological environment and influences EV target cell interaction and function. EVs isolated from human placental expanded stromal cells (PLX) by tangential flow filtration (TFF) have an intact protein corona, which can be visualized by super-resolution microscopy and transmission electron microscopy, providing pro-angiogenic activities. However, when PLX-EVs were precipitated by ultracentrifugation or size exclusion chromatography, the protein corona was affected and proangiogenic activities were abolished. Intriguingly, the EV protein corona could be reconstituted by adding defined pro-angiogenic corona components, which also restored the ability of these EVs to promote *in vitro* angiogenesis^[5]^. These findings imply that molecules contributing to the EV corona importantly contribute to EV functions. Furthermore, EV isolation procedures may affect the integrity of the corona and alter EV functionalities.

Next, Jochen Hernadez Bücher (MPI for Medical Research, Heidelberg, Germany) explored whether cells employ the presentation of ligands on secreted EVs to enhance their signaling properties (described by the term *vesicle-induced receptor sequestration*). Synthetic EVs that were engineered to expose ligands such as FasL and RANK on their surface indeed transmitted their signals more than 100-fold more efficiently than non-EV-bound ligands. Based on diffusion simulations using the immunological synapse as a model, the authors proposed a universal mechanism by which EVs allow for the concentration of ligands promoting local aggregation of membrane receptors facilitating signaling at the EV-cell interface. This could represent a unique structural profit of EV-mediated signaling.

The remaining two scientific presentations of the session focused on the biogenesis of small EVs (sEVs), especially of exosomes within the late endosomal compartment. Kerstin Menck (University Hospital Münster, Germany) outlined the role of the adapter protein CD2AP during different steps of sEV biogenesis and cargo sorting. Interfering with CD2AP expression in a human breast cancer cell line resulted in reduced levels of CD63 and Syntenin in sEVs recovered from the culture supernatant. Moreover, CD2AP overexpression increased CD63 and Syntenin levels in sEVs. Analysis of the endosomal trafficking of CD63 revealed that CD2AP knockdown was accompanied by an accumulation of CD63 in LAMP2-positive compartments, indicating that CD2AP assists CD63 trafficking through this compartment. Furthermore, knockdown of CD2AP diminished the presence of CD63 and Syntenin in large artificial endosomes generated by overexpression of a constitutive active form of the GTPase Rab5 (Rab5-Q79L), while overexpression had the opposite effect. CD2AP is known to directly interact with Alix and thus may act as an adaptor protein for the selection of cargo proteins of exosomes generated in the Syntenin-Alix pathway. Consistently, experimental reduction of Alix expression counteracted the CD2AP functions observed in exosome biogenesis. For her excellent talk at the symposium, Kerstin Menck was awarded with the Margot Zöller Prize.

Barnabas Irmer (University Hospital Münster, Germany) presented a proteomic profiling approach to identify Syntenin-dependent sEV cargoes. Since Syntenin is overexpressed in different types of tumors, Syntenin-dependent modulation of the EV cargo may have functional effects contributing to tumor pathology. sEVs collected from Syntenin-knockout cells revealed a considerable difference in their protein cargo when compared with wild-type EVs, while the cargo of large (l) EVs was similar. Proteins downregulated in Syntenin-deficient sEVs were largely associated with processes of cell-adhesion, extracellular matrix assembly, and cell-cell contacts. Notably, the tumor-antigen-epithelial cell-adhesion molecule (EpCAM) was shown to directly bind to Syntenin by surface plasmon resonance spectroscopy and, consequently, was also lacking in Syntenin-deficient sEVs. Thus, Syntenin levels affect sEV cargo including loading with adhesion receptors, which may modulate the tropism and other signaling functions of tumor cell-derived sEVs.

In summary, the lesson that we may have learnt from this session is that the dynamics and diversity of EVs, which puzzled us from the early days of EV research, is now being unraveled at the molecular and functional level. Deciphering the mechanisms regulating EV cargo composition, whether inside EVs, presented on the membrane, or associated with the surface in form of the corona, are key to understanding EV signaling functions.

The session closed with two talks from industrial sponsors, providing insight into recent technological developments. Siobhan King (ONI/Oxford Nanoimaging Ltd, UK) presented a high-resolution microscopy platform, a dSTORM device, allowing characterization of EV composition and EV heterogeneity at the single molecule level. She also presented a newly developed software platform providing a set of different analysis and quantification tools. Aquiles Carattino (Dispertech B.V., NL) introduced a system for measurement of both size and scattering intensity of single EVs at a high resolution within the size range of 20-150 nm. The system provides a convenient table-top technology for measuring single EVs as it works label-free, requires only 5 µl of sample volume, and appears to deliver accurate and reproducible results with biological samples.

## ENABLING TECHNOLOGIES: REFERENCE MATERIALS, PURIFICATION AND ANALYSIS

The second session focused on innovative technologies allowing EV analyses and preparation and was moderated by Michael W. Pfaffl and Jan Lueddecke. The session was opened by the keynote lecture from Marcella Chiari from the Institute of Chemical and Technological Sciences of the National Research Council of Italy. She presented her recent developments using integrated systems for the isolation and later characterization of EVs from a broad spectrum of matrices or liquid biopsies. Thus far, EVs have prospective potential in the medical field, but there is no consensus about the “best and most integer” isolation method to receive intact and functional EVs. Within two EU-funded HORIZON 2020 projects [INDEX (integrated nanoparticle isolation and detection system for complete on-chip analysis of exosomes) and MARVEL (evolving reversible immunocapture by membrane sensing peptides towards scalable EV isolation)], new separation methods for larger and smaller EVs from cell culture supernatants and complex biofluids were developed by Marcella’s group. She described a novel approach for affinity isolation and *in situ* enrichment of EVs from plasma allowing separation of concentrated, pure, and intact EVs. The released EVs were subjected to a multi-parameter interferometric analysis providing information on vesicle size, number, and phenotype. Based on single-stranded DNA tagged antibodies, EVs were captured on the surface of complementary DNA strand-loaded magnetic beads. Following binding of the EVs to the bead surface, they can be released from the beads by DNase I digestion. Using this immunocapture strategy, retained EVs were prepared from complex biological fluids. The conditions were mild, such that they preserved the EVs’ integrity and functionality. Furthermore, the captured EVs can be characterized by various methods (nanoparticle tracking analysis, electron microscopy, and flow cytometry). Furthermore, Marcella presented a second new EV capturing method that binds EVs directly to a surface of microchips. This method is based on a membrane sensing peptide that serves as a highly efficient ligand independent of the composition of EV surface proteins. In particular, bradykinin-derived peptide baits were used to capture EVs on biological matrices. Captured EVs were characterized by single particle counting and sizing. Furthermore, their CD9, CD63, and CD81 content was analyzed with fluorescent anti-CD9, anti-CD63. and anti-CD81 antibodies, respectively.

The second talk was presented by Christian Preußer from the Institute for tumor Immunology located at the Philipps University in Marburg. He is head of the newly established and first EV Core Facility in Germany^[6]^. The scientific goal of the core facility is to interface various scientific research groups working with EVs at the medical campus , unravel EV-mediated communication processes within the tumor microenvironment, and demonstrate their impact on tumor development and progression. Christian presented the actual methodological portfolio and techniques implemented in the last months and the near future. Thus far, various techniques being used in EV research have been optimized and are now successfully used in the facility. Additional technologies considered as powerful tools in EV research will be further included. Christian elaborated on free flow electrophoresis (FFE), a technology being provided by FFE Service (Feldkirchen, Germany), a well-established semi-preparative method to separate and prepare analytes, e.g., by inherent differences of their electric charges. FFE combines a flow driven longitudinal transport of sample material with vertical electrophoresis and allows separation of sample components into up to 96 different fractions. Protocols are currently optimized for the EV preparation and characterization in a collaborative approach among FFE Service, the core facility in Marburg, and the group of Bernd Giebel in Essen. FFE appears suitable for the purification and characterization of EVs from all types of biofluids or bacteria. Furthermore, the EV Core Facility offers state-of-the-art techniques, such as single vesicle characterization and phenotyping. Through networking with other omics core facilities at the campus, an in-depth and complex molecular characterization of EVs can be offered.

During the remainder of the session, talks were given by young scientists, presenting their PhD or postdoc projects. Yanis Mouloud from the Institute for Transfusion Medicine of the University Hospital Essen introduced a novel, TERT-based immortalization strategy that allows immortalization of human mesenchymal stromal cells (MSCs) for the scaled MSC-EV production. Using this technology, Yanis raised different MSC lines at the clonal level. Connected to their immunomodulatory properties, MSC-EVs are considered as therapeutic agents for many diseases. Previously, his group conducted the world’s first MSC-EV application in a human; they successfully treated an otherwise treatment-refractory graft-*vs.*-host disease (GvHD) patient with a self-produced MSC-EV preparation^[7]^ and confirmed the therapeutic potential of MSC-EV products in several animal models. Observing significant variations in the immunomodulatory activity of independent MSC-EV preparations, the clonally expanded immortalized MSCs (ciMSCs) should help avoid product heterogeneities arising from the usage of MSCs of different donors and replicative senescence processes of primary MSCs. Yanis demonstrated that his ciMSC-EV products retain the immunomodulatory potential that the group originally described for EV products of primary MSCs. Currently, ciMSC-EV products are being tested in various disease models. Ideally, in the future, ciMSCs can be used for the scaled-manufacturing of therapeutic *off-the-shelf* MSC-EV products for clinical application.

In the following talk, a new EV isolation technique was presented by Dapi Menglin Chiang working at the Department of Animal Physiology and Immunology, the School of Life Sciences, Technical University of Munich in Freising Weihenstephan. EV isolation is a time-consuming process and is often accompanied with a high amount of lipoprotein contamination. Hence, a fast isolation method with high purity of functional EVs is urgently needed, among others, for various diagnostic and clinical applications. Furthermore, the functional properties of the isolated and contamination free EVs should be maintained. The new EV isolation via EXÖBead® is functionally based on a galectin-glycan recognition. EXÖBead® are magnetic beads coated with galectins that bind EV glycan residues with high affinity. Furthermore, EVs can be easily eluted functionally intact from beads by high concentration of lactose. Isolated EVs showed high abundance of specific EV markers such as CD9, CD63, CD81, and TSG101 in flow cytometry. Compared with other isolation methods, such as size exclusion chromatography (SEC), EXÖBead® isolation EVs resulted in low apolipoprotein A1 contamination. To prove the clinical application, Dapi and his team isolated and analyzed tumor-derived plasma EVs (TDEs) from patients suffering from head and neck squamous cell carcinoma (HNSCC). TDEs are involved in the HNSCC pathogenesis, such as tumor progression and immune evasion. In the clinical samples, HNSCC biomarkers such as EpCAM, PanCK, and PD-L1 were identified on CD45- plasma-derived EVs. Significant difference of TDE immune suppression ability were observed, compared to a classical polyethylene glycol isolation (PEG; ExoQuick®). Furthermore, the EV functionality of the EXÖBead® could be shown in a peripheral blood mononuclear cells activation assay. In summary, the new EXÖBead® isolation is an easy-to-use method with low lipoprotein contamination that maintains the functional abilities of EVs.

The focus of the next presentation, delivered by Josepha Roerig from the Institute of Pharmacy at the Medical Faculty Leipzig, was the uptake and transport of milk-derived EVs across an intestinal barrier model via Caco-2 cells. In recent publications, bovine milk-derived EVs are described as promising oral drug delivery systems, since they withstand the harsh conditions of the gastrointestinal tract. However, the key factors regarding the milk EV uptake and ability to pass the intestinal barrier is still not understood. The presenter addressed these aspects by *in vitro* standardization approaches. Milk EVs were enriched using differential centrifugation followed by SEC and characterization according to the MISEV 2018 guidelines, using cryo transmission electron microscopy (Cryo-TEM), nanoparticle tracking analysis (NTA), and numerous EV marker proteins. Uptake properties of milk EVs were compared to liposomes in Caco-2 cell culture. Three different EV labeling technologies were benchmarked to obtain reliable and stable EV staining results. Milk EVs outperformed liposomes regarding uptake efficiencies. Furthermore, confocal microscopy revealed the internalization of milk EVs, whereas liposomes rather remained attached on the Caco-2 cell surface. Josepha and coworkers provided the foundation for future investigations of milk EVs for oral drug delivery purposes and for the appropriate comparison with liposomes.

The last talk in this session was given by Hui-Yu Liu working at the Institute of Nanotechnology at Karlsruhe Institute of Technology. Her topic was a new isolation technique using tailored lipid membranes to capture EVs. She presented the use of antibody-functionalized lipid patch arrays for rapid, highly selective and sensitive detection of EVs from complex media. Scanning probe lithography techniques offer unique opportunities for highly localized chemical surface functionalization to bind biomolecules, e.g., tailored supported lipid membranes (SLM). The SLM obtained are single- to multilayer stacks of biomimetic phospholipid membranes that can be tailored for shape and function by the printing process and admixing of functional lipid compounds. This allows, e.g., to alter charge or mechanics of the membranes or to introduce selective binding sites for the capture or presentation of antibodies, e.g., to bind EVs. By exploiting the natural process of vesicle fusion, the use of SLM patch arrays can greatly facilitate the EV detection process and retain the information on the array for downstream analysis.

The session was completed by two industrial talks presenting their latest technologies for EV isolation and characterization. Ingrid Bloß from Particle Metrix GmbH presented results from colocalization experiments using a novel multi-fluorescence NTA device (f-NTA). The fluorescently labeled EVs can now be specifically tracked and will provide a deeper and more specific information, e.g., the numbers of EVs containing CD63, CD81, CD9, and CD41 antigens.

The second industry talk was presented by Stephane Mazlan from Izon Science Europe Ltd. He introduced an automatized process for the EV isolation and single particle characterization via tunable resistive pulse sensing (TRPS). This technology has demonstrated precision in both size and concentration determination where subpopulations in multimodal samples can be accurately portrayed and distinguished. Mazlan reported the current TRPS instrument can measure EVs as small as 30 nm (Exoid, Izon Science) and offers the possibility for automation.

## PHYSIOLOGICAL AND PATHOPHYSIOLOGICAL ASPECTS OF EVS

The session addressed basic questions regarding the nature and purpose of EV release and their role in intercellular communication processes, and how specific cargo loading is regulated. Tumor-released EVs contribute to almost all hallmarks of cancer and the central topic of the session raised their role in cancer initiation and progression. Cutting edge data including their impact on metastasis, central signaling pathways (Toll-like receptor signaling), the extracellular matrix or tumor-associated bystander cells were discussed. Moreover, data on ciliary-associated EVs and their impact on the tumor associated WNT signaling pathway were presented. 

The guest speaker Ralf Jacob from the Philipps University Marburg presented the latest results in his ongoing research concerning the EV-associated release of the β-galactoside-binding galectin-3. Galectin-3 is a lectin that contributes to tumor microenvironment immunosuppression, and it regulates diverse functions including cellular homeostasis and cancer biology. Of note, in epithelial cells, the apical transport occurs via different pathways, and galectin-3 was identified as a sorting receptor to recruit cargo molecules into apical transport vesicles. Ralf’s group discovered that a late domain motif in the N-terminal domain of galectin-3 directs the recruitment of the lectin into multivesicular bodies for non-classical exosomal secretion into the apical milieu. This highly conserved P(S/T)AP domain motif is required for the interaction with the ESCRT-I factor Tsg101. Tsg101 is critically involved in ESCRT-regulated endosomal membrane remodeling, enabling vesicle formation and finally their release as exosomes. Once at the apical membrane, galectin-3 reenters the cell by endocytosis and traverses the endosomal pathway to be recycled back to the cell surface. This recycling is modulated by the pH gradient between the extracellular milieu and endosomal organelles.

The session went on with Lothar C. Dieterich from the Swiss Federal Institute (ETH) of Technology in Zurich who presented his findings on how pre-metastatic niches are formed in part by melanoma-derived EVs. He showed that melanoma EVs shuttle tumor antigens via the lymphatic vessels to tumor-draining lymph nodes. Here, cross-presentation of the antigens to the antigen-specific CD8^+^ T cells leads to enhanced apoptosis of the immune cells and thus an immunosuppressive phenotype and tumor progression. Lothar pointed out that the EV-dependent cross-talk of lymph nodes and lymphatic endothelial cells represents a novel pathway contributing to melanoma progression and might serve as a new target for melanoma therapy. For this excellent presentation, Lothar C. Dieterich was awarded with the Margot Zöller Prize.

Next, María Gómez-Serrano from the Institute of Tumor Biology in Marburg presented her newly established *ex vivo* system to analyze EVs of ovarian cancer-associated adipocytes (CAAs). The role of tumor-associated adipocyte-derived EVs in tumor biology is largely enigmatic, which is also due to the lack of established methods to collect and analyze these EVs. To tackle this issue, Gómez-Serrano used primary material, i.e., human mentum, which was cultivated in the presence of ascites, the characteristic tumor microenvironmental fluid of ovarian cancer. During the reprogramming of adipocytes to CAAs in this system, the adipocyte/CAA-released EVs were collected, purified, and characterized. Preliminary data indicate an increase in the number of released EVs, a shift in the average size of EVs, and a change in the percentage of CD63^+^ EVs comparing adipocyte and CCA-released EVs. Their cargo and modulatory effect on tumor and host cells is currently under investigation.

Primary cilia and cilia-EVs were the focus of the presentation of Ann-Kathrin Volz from the Institute of Molecular Physiology Johannes Gutenberg University in Mainz. Comparing cilia-deficient Bardet-Biedl syndrome (BBS) mutant cells and wild-type cells, she observed an increased release of small EVs by BBS cells and an altered cargo composition. The EVs of BBS mutants were enriched with miRNAs and proteins associated with WNT signaling - a protein family crucial for developmental and disease processes^[8]^. Of note, knock down of dicer, an enzyme critically involved in miRNA processing, did not show any effect on the WNT-modulating activity of resulting EVs. This suggests that miRNAs are not essential in this pathway, but a protein-mediated mechanism may be involved that will be unraveled in future studies to gain new insights into ciliopathy disease pathogenesis.

Meike J. Saul from the Department of Biology, Technical University of Darmstadt presented her findings on an EV-mediated feedback loop concerning prostaglandin E2 (PGE2). She reported that PGE2 induces the sorting of miR-574-5p into small EVs in two lung cancer cell lines. The miRNA then activates Toll-like receptors that in turn lead to a decrease of PGE2. In contrast, intracellular miR-574-5p acts oppositely and induces PGE2 synthesis. Thereby, intracellular and extravesicular-derived miR-574-5p regulate the PGE2 protein level in lung adenocarcinoma. Interestingly, this was only observed in adeno- but not in squamous cell carcinoma, indicating a cell-specific response which is probably dependent on the unique tetraspanin composition of the studied cell types.

The next speaker, Venkatesh Sadananda Rao from the Department of Visceral, Thoracic and vascular Surgery, Dresden University of Technology, elucidated the role of extravesicular tissue inhibitor of matrix metalloproteinase-1 (TIMP1) in colorectal cancer (CRC) and colorectal liver metastasis samples during invasion and metastasis. Using 3D extracellular matrix (ECM) remodeling assays, he showed that primary fibroblasts in the presence of CRC-EVs induced ECM remodeling, which was abrogated when TIMP1 was inhibited. This mechanism seems to be clinically highly relevant since the TIMP1 level in the invasive front of liver metastasis tissue samples was positively associated with a poor progression-free survival of the patients. Thus, targeting TIMP1 may represent a novel approach to prevent or attenuate the formation of liver metastasis.

The last speaker of this session was Samantha Lasser from the Skin Cancer Unit located at the German Cancer Research Center (DKFZ) in Heidelberg. She investigated the role of melanoma-derived EVs and with them a group of miRNAs that mediate the conversion of normal human monocytes to myeloid-derived suppressor cells. In a murine system, she analyzed the role of miR-125a on immature myeloid cells. She observed an upregulation of immunosuppressive features such as PD-L1 and factors of the Toll-like receptor family, NF-κB, and JAK-STAT families. To further elucidate the combined effect of EV-associated miR on different Toll-like receptors and finally on antitumor immunity, Lasser plans to use immature myeloid cells from mice with knockouts for specific Toll-like receptors.

In summary, the researchers of this third session presented many different aspects of the formation, promotion, and progression of various malignancies and the important roles EVs seem to play in them.

## EV IN CLINICAL DIAGNOSTICS - PERFORMING RELIABLE EV BIOMARKER ANALYSIS

The robust detection and analysis of EVs is an important technical challenge during their development as biomarkers. Enhanced rigor is required to rule out any unwanted byproduct detection and verify that EVs during diseases states are substantially different from those under physiological conditions. Marca Wauben from Utrecht University, Department of Biomolecular Health Science opened the present session on EV biomarker assessment for clinical diagnostics. Marca is a well-known pioneer in the field of single EV analysis by flow cytometry. In her talk, she clearly pointed out that high-resolution evaluation of EVs by flow cytometry is needed and challenging. Interestingly, there are substantial inter-platform and inter-laboratory differences in the study of EVs. That is, two labs may not be able to identify comparable EV populations using comparable instruments. These finding underline the need for standardization of EV isolation protocols and methodologies and are discussed in an international expert consortium, which has already provided a guideline for flow cytometry based-EV analyses^[9]^. Adding another layer of complexity, Marca also discussed the overlap of EV analysis with lipoprotein particles and other protein complexes from human samples.

This presentation was followed by several talks from young scientists at the PhD and postdoc level. Jerome Nouvel from the Institute of Infection Prevention and Hospital Epidemiology, University of Freiburg presented the validation of a fast protein liquid chromatography for EV isolation from blood specimens. The method was nicely combined with established size exclusion chromatography using alternative gel separation matrices. It was shown that separation of EVs from serum-derived lipoproteins was feasible using this method. In a subsequent step, this technique was applied to robustly isolate EVs from pancreatic cancer patients. A consecutive analysis of the tumor EVs is still needed, but the fast protein liquid chromatography method was reliably established.

Focusing on a different aspect of EV release, Elmo Neuberger from University of Mainz, Department of Sports Medicine, Prevention & Rehabilitation reported on the influence of physical exercise on DNA being associated with EVs. While it was confirmed that cancer cell EVs could carry DNA as a cargo, it appears less investigated whether under different physiological conditions “freely circulating” DNA is associated with EVs. For the investigation, healthy volunteers underwent treadmill exercise, and their EVs and related DNA were isolated from the volunteer’s blood plasma and studied in more detail. For the removal of non-EV-associated, co-purified free circulating DNA, obtained EV samples were treated with DNase. According to the results presented, only 0.12% of the plasma DNA is associated with EVs, approximately a fifth of it being encapsulated as luminal EV cargo. While the amount of EVs as well as total EV-associated DNA increased with exercise, luminal DNA cargo did not increase within the prepared EV fraction. Thus, EVs released under physical stress conditions such as exercise are increasingly decorated with DNA without increasing their luminal DNA cargo^[10]^.

Following this presentation, two postdocs from the Friedrich-Alexander University Clinics Erlangen - **Jan van Deun** and **Jan-Ole Bauer **- presented their joint work on EVs as biomarkers in the detection of tumor and inflammation. van Deun presented that melanoma patients carry protease-harboring EVs which are major players in inflammatory processes. A method was established to rapidly detect the presence of these proteases in a cohort of healthy volunteers and melanoma patients. Protease activity in these samples was studied using FRET-based substrates, which helped to identify 14 proteases predominantly identified in the group of melanoma patients. When a neural network-based classification model was applied, van Deun showed that a < 85% specificity and sensitivity in the identification of tumor patients was achieved. These results provide a sound basis for further biomarker establishment in melanoma patients. In addition to these findings, Bauer was able to correlate EV-associated protease activity to inflammatory effects in a larger group of healthy volunteers. While the majority of these volunteers did not show any elevated levels of protease activity, approximately 30% had higher inflammatory state; this was most likely due to a previous SARS-CoV-2 vaccination. It was concluded that, although EV-protease activity could reflect inflammatory dispositions in patients, a substantial number of healthy volunteers also showed higher protease activity, findings that require larger clinical cohorts in the future.

Maike Saul presented the work of her PhD student Kai Breitwieser (Department of Biology, Technical University Darmstadt) focusing on small EVs and their potential for biomarker development. Although several tetraspanins, including CD63 and CD81, are well established for confirming EV identity, their abundance and distribution seems to vary between EV source and populations. To address this variation, a detailed expression and colocalization study was conducted using a microchip-based detection technique (ExoView®). This method allows for purification free assessment of marker density and relation, and it may potentially be suitable for future EV-biomarker establishment.

This scientific session was closed by Karolina Soroczyńska, a PhD student from the Department of Biochemistry, Medical University of Warsaw (Poland). She is studying endometriosis, a gynecological disorder in the female reproductive system for which clinical therapies are yet lacking. It was previously found that patients showed a higher concentration of two subtypes of arginase enzymes. Her group became subsequently interested in establishing a relationship with small EV release in the patients concerned. Indeed, she demonstrated the presence of arginase in EV preparations, most of the enzyme recovered on the EV surface. A slight increase in EVs number for affected patients was detected compared to healthy controls. Overall, such arginase-positive EVs may influence endometriosis progression and require future investigations.

Finally, the session was concluded by presentations from the industrial partners. Ben Peacock from NanoFCM presented a nano-flow cytometer, which allows both fluorescence and side scatter detection of small particles, as well as simultaneous sizing and identification of subpopulations. Andrew Malloy from NanoView Biosciences introduced a chip-based system that captures EVs via specific antibodies and allows sizing and fluorescent antibody-based characterization of individual captured Evs. Peter Rhein from Luminex introduced imaging flow cytometer platforms, i.e., the AMNIS image stream and AMNIS cell stream, both allowing high-resolution camera-based detection of objects of different sizes including cells and small EVs. 

## DRUG DELIVERY AND THERAPEUTIC APPROACHES

At least a proportion of EVs mediate intercellular communication at local and distant sites, likely in a specific manner. Apparently, EVs contain tropisms to selected target cells and tissues. Coupled to their role in physiological and pathological processes, EVs prepared from appropriate cell types now provide the chance to manipulate such processes in a very effective manner. Consequently, EVs have emerged as a promising tool for novel therapeutic interventions. On the one hand, native EVs such as the aforementioned MSC-EVs are considered as novel therapeutic agents for many diseases. On the other hand, coupled to their tropism, EVs are envisioned as natural drug delivery vehicles^[11]^, in which big pharma have become very interested. To use EVs as drug delivery vehicles, strategies need to be developed in which EVs can be manipulated and loaded in very controlled and efficient manners. A pioneer in this area is Prof. Samir el Andaloussi heading a group at the Department of Laboratory Medicine in the Clinical Research Center of the Karolinska Institutet in Stockholm (Sweden). He is also one of the three co-founders of the emerging EV company EVOX Therapeutics Ltd. In his keynote lecture, el Andaloussi introduced in this session, which was chaired by Oskar Staufer and Bernd Giebel, some of the latest results of his group, which develops genetic engineering strategies for EVs permitting loading of therapeutic proteins and RNAs as well as displayed peptides and proteins for targeted delivery to EVs that should serve as biotherapeutics. At first, el Andaloussi described the principles of endogenous EV engineering and the use of various constructs in which luciferase (Luc) is fused to annotated EV proteins/domains for the identification of proteins/domains allowing very effective protein cargo loading to EVs. For the example of tetraspanin-Luc fusion proteins, he demonstrated how such fusion proteins can be used for assessing pharmacodynamics and pharmacokinetics of engineered EVs in mice. Regularly, administered EVs distribute quickly in various tissues and reveal short half-life times, especially in the plasma. Upon introducing engineered forms of a tetraspanin construct containing albumin-binding domains (ABDs) in the second outer loop domain of the given tetraspanin, the half-life time of resulting EVs could be substantially increased; consequently, much higher EV amounts could be delivered to tissues including tumors and lymph nodes. In another engineering strategy, his group introduced an Fc-binding domain into the second outer loop domain of the given tetraspanin and can now load EVs with any given antibody. For the examples of anti-PD-L1 and anti-HER2 antibody-loaded EVs, el Andaloussi demonstrated that such EVs bind to cells expressing the respective antigens and are subsequently taken up by these cells. More precisely, he showed the antibody mediated EV uptake in B16F10 melanoma and SKBR3 breast cancer cells and reported that, following i.v. administration of such EVs, being additionally loaded with chemotherapeutic agents, they specifically target and eliminate subcutaneously implanted melanoma cells, thereby significantly improving the survival rate of the respective mice. The last part of his presentation covered the topic of decoy EVs, EVs that are engineered to display signal incompetent receptors on the EV surface being used to sequester disease-causing soluble factors in blood. el Andaloussi showed a proof-of-principle experiment in which TNFR or GP130 was displayed on EVs to inhibit inflammation promoting TNF or IL6 signaling, respectively, and how this strategy could be improved upon using N-terminal Syntenin tethered to the respective receptors and by the use of luminal multimerization domains to enhance receptor clustering. Applying this strategy, el Andaloussi’s group already successfully used this decoy strategy in three different disease mouse models: in an LPS-induced sepsis model, a TNBS-induced IBD colitis model mimicking Crohn’s disease, and an EAE multiple sclerosis model. Importantly, he finally showed that the engineering strategy can be used to generate so-called double decoys where single EVs display both receptors for highly efficient treatment of colitis, surpassing the effect of conventionally used biologics.

Following this outstanding lecture, Aladin Samara of the Felsenstein Medical Research Center of the Rabin Medical Center in Petah Tikva in Israel reported about the concept in using NK-derived EVs for leukemia treatment. The group had prepared small EVs from conditioned media of the NK92MI cell line and explored their killing potential in some *in vitro* experiments*.* They improved time- and dose-dependent killing activities in all leukemic cell lines tested and observed a substantial reduction of colony formation potentials of patient-derived leukemia cells cultured in the presence of the given EVs. In contrast, these NK-EVs did not affect healthy B cells. The killing effect was correlated with their uptake; labeled NK-EVs were effectively taken up by leukemic cells but not by healthy B cells. Next, intending to develop off-the-shelf NK-EV products for leukemia treatment, Aladin’s team wants to test the anti-leukemic potential of the NK-EVs in a xenograft leukemic mouse model*. *

The third talk in this session was given by Katrin Bedenbender of the Institute for Lung Research of the Philipps-University Marburg. Katrin reported about the impact of bacterial EVs and secreted host factors on the function of endothelial Ribonuclease 1 (RNase1) in sepsis. In addition to their importance for vascular homeostasis, endothelial cells are essential sensors for pathogens, inflammatory mediators and immunity. Once being activated. amongst others, endothelial cells secrete the vessel protective factor RNase1. However, massive and persistent inflammatory conditions frequently result in RNase1 repression, often being associated with vascular pathologies including sepsis progression. In her project, Katrin investigated whether bacterial EVs, so so-called outer membrane vesicles (OMVs), of different bacteria, i.e., *Escherichia coli*, *Klebsiella pneumoniae,* and *Salmonella enterica* from serovar *Typhimurium*, affect the endothelial RNase1 function. She reported that applied OMVs from sepsis sepsis-inducing bacteria in contrast to non-sepsis sepsis-inducing bacteria significantly reduced the activity of RNase1 and promoted endothelial cell activation, as indicated by increased IL-8, CXCL10, ICAM-1, and IL-1β expression. Notably, the LPS-neutralizing antibiotic polymyxin B was found to effectively prevent both, the OMV-induced RNase1 repression as well asnd the endothelial cell activation by the prevention of Toll-like receptor 4 (TLR4) activation. Thus, the current results imply that, via TLR4 activation, OMVs of sepsis sepsis-inducing bacteria promote endothelial cell activation and thereby contribute to the disease progression. Aiming to develop novel therapeutic anti-sepsis strategies, the underlying signallingsignaling cascade should be investigated in more detail. 

Finally, two industry talks were given. Vi Tran from FUJIFILM Wako Chemicals Europe GmbH presented the increasing product portfolio the company is providinges for EV research, including products for EV purification and detection. Thomas Bauer-Jazayeri of Fidabio introduced a new flow-induced dispersion analysis device that can be used for EV characterization. The device enables measurements of binding affinity and size (absolute in nm) of biomolecules including EVs in solution, e.g., in storage buffers or their original bioliquids.

## MISEV GUIDELINES, CONTROLS, MAIN PITFALLS IN EV RESEARCH: INTRODUCTION AND ROUND TABLE DISCUSSION

The last session of the meeting chaired by Irina Nazarenko started with a presentation by Dirk Strunk (Paracelsus University Salzburg), who started by asking the audience about their handling and respecting of the MISEV guidelines^[12]^. Then, he reported on a systematic text-mining approach in which his group assessed for the adherence of published EV studies to the MISEV2018 criteria^[13]^. The analysis revealed that over the years an increasing proportion of EV manuscripts refers to and respects the MISEV criteria. Interestingly, studies including a more elaborated MISEV-guided methodology were cited with higher frequency. Apparently, the MISEV guidelines are successfully helping to improve the quality of EV research. Dirk’s presentation was followed by a roundtable discussion on the importance of the MISEV criteria for the progression of the EV field but also about their limitations. The group of invited senior EV researchers and the session chairs provided their expertise of how to apply and deal with these criteria and answered specific questions of the audience regarding the limitations of the field. Condensing this session’s take home message, it became apparent that common efforts are required to push the technical limitations in the EV field. EV research in many aspects is still in a developing phase and for its ongoing progress requires a community-driven reflection. Approaches of the international EV community to define guidelines such as MISEV2018 help to sharpen our overall understanding and provide recommendations for everyday research to the profit of overall quality of EV research. However, we also need to be aware that within the progressing EV field these recommendations are continuously evolving, sometimes challenging existing recommendations. Thus, it is an ongoing international goal to dynamically improve and update existing MISEV criteria. Indeed, currently, a new version is being prepared, which is expected to be published in summer 2022. Since limitations and required changes are discussed among EV experts in advance and many GSEV members participate in such discussions, it is always a good idea that scientists entering the EV field discuss with EV experts upfront to avoid some unnecessary pitfalls, which sometimes are hard to recognize within the overwhelming flood of literature about EVs. GSEV is dedicated to providing an interdisciplinary and up-to-date platform allowing the information exchange among its members and is open to all scientists interested in EV research, both within and outside Germany.

## POSTER SESSION

Within the poster session, 27 posters were shown and presented mainly by young scientists. It was a huge challenge in selecting the “right” posters for the Peter Altevogt Prize. Their topics of the posters covered the whole area from basic to clinical research and addressed the origin and biogenesis of EVs, the methodology of EV purification and analysis, and the role of EVs as biomarkers and therapeutic agents in (patho-)physiological processes. The poster voting committee, the group of all orally presenting young scientists, finally reached a decision by swarm intelligence and selected three “Best Posters”:

Fang Cheng Wong from the University Hospital Dresden won the third prize for her research on the EV-mediated crosstalk between pancreatic cancer and Schwann cells. Pancreatic cancer is characterized by its capacity to invade the neural system, especially Schwann cells. In both murine and human 3D culture models, Fang Cheng Wong showed that the migratory potential of Schwann cells increases after treatment with pancreatic cancer cell-derived EVs but not with EVs from non-cancer cells. The effect was abrogated in the presence of EV uptake inhibitors such as heparin and EIPA, indicating a critical role of EVs for the promotion of pancreatic cancer invasion. 

The second prize was awarded to Moshin Shafiq from the University Medical Center Hamburg-Eppendorf for his research on EVs and their role in Alzheimer’s disease (AD). He investigated the cellular prion protein (PrPC), a protein abundantly expressed on the cell surface as well as on EVs (exosomal PrCP). Using wild-type and PrPC-deficient EVs from neuroblastoma cells, Moshin analyzed Aβ fibril formation, a characteristic feature of AD. He detected an altered proteomic and lipidomic profile between the two samples and the strong sequestration of Aβ in wild-type EVs compared to the KO, thus highlighting the crucial role of PrPC-carrying EVs in the pathophysiological processes of AD.

Finally, the first prize was awarded to André Cronemberger Andrade from the Paracelsus Medical University in Salzburg (Austria) for his research on the role of EVs in angiogenesis. Using different purification methods (TFF and ultracentrifugation), he prepared EVs from induced pluripotent stem cells cultured under various hypoxic conditions and analyzed their angiogenesis potential. He showed that hypoxia, in general, led to the stabilization of EV cargoed HIF-1α, while the purification method specifically influenced the enrichment of tetraspanins and Alix in obtained EV preparations. The pro-angiogenic effect was reduced when extended purification protocols were used and correlated with a reduced VEGF content in respective EV samples, again highlighting the impact of the EV preparation method on the activity of obtained EV products.

At this point, the authors like to congratulate all the winners of the Margot Zöller and Peter Altevogt Prizes and thank all speakers, poster presenters, the industry representatives, and all other participants for an outstanding meeting.

## CONCLUSION

Overall, the GSEV autumn meeting 2021 provided a highly productive platform for interactions among the participants, especially for the German EV young scientist community. The talk and poster sessions were accompanied by intense discussions, which carried into the coffee breaks and beyond. Advances reported related to the specific molecular and functional characterization of EVs and the refinement of strategies for therapeutic applications of EVs. EV science continues to advance through focused technology development, consistent research guidelines that are accepted across the community, and regular exchanges between basic and applied EV scientists. The establishment of future collaborative research centers with a focus on EV science and the implementation of EV core facilities, such as at the University of Marburg, make a decisive contribution to promoting excellent EV science in Germany and worldwide.

With its next generation of motivated and educated EV researchers, Germany appears to be well equipped for the challenges in this area. The GSEV board on behalf of the whole society thanks all contributors, the sponsors, and the organizing team for their efforts making this meeting a success. We are all looking forward to our next meeting organized together with the IGLD, taking place from March 10th-12th 2022 in Frankfurt am Main. The meeting is completely free of registration fees and the program will address exciting topics, from techniques to EV functions and regulatory affairs. Mark your calendar and see you in March!

## DECLARATIONS

### Copyright

© The Author(s) 2021.
